# Genome-wide identification of *WRKY* family genes and their response to cold stress in *Vitis vinifera*

**DOI:** 10.1186/1471-2229-14-103

**Published:** 2014-04-22

**Authors:** Lina Wang, Wei Zhu, Linchuan Fang, Xiaoming Sun, Lingye Su, Zhenchang Liang, Nian Wang, Jason P Londo, Shaohua Li, Haiping Xin

**Affiliations:** 1 Key Laboratory of Plant Germplasm Enhancement and Specialty Agriculture, Wuhan Botanical Garden, The Chinese Academy of Sciences, Wuhan, PR China; 2 Beijing Key Laboratory of Grape Sciences and Enology, Laboratory of Plant Resources, Institute of Botany, The Chinese Academy of Sciences, Beijing, PR China; 3 University of Chinese Academy of Sciences, Beijing, PR China; 4 United States Department of Agriculture–Agriculture Research Service, Grape Genetics Research Unit, Geneva, NY, USA

**Keywords:** WRKY transcription factor family, Grapevine, Biotic and abiotic stress, Cold stress

## Abstract

**Background:**

*WRKY* transcription factors are one of the largest families of transcriptional regulators in plants. *WRKY* genes are not only found to play significant roles in biotic and abiotic stress response, but also regulate growth and development. Grapevine (*Vitis vinifera*) production is largely limited by stressful climate conditions such as cold stress and the role of *WRKY* genes in the survival of grapevine under these conditions remains unknown.

**Results:**

We identified a total of 59 *VvWRKYs* from the *V. vinifera* genome*,* belonging to four subgroups according to conserved WRKY domains and zinc-finger structure. The majority of *VvWRKYs* were expressed in more than one tissue among the 7 tissues examined which included young leaves, mature leaves, tendril, stem apex, root, young fruits and ripe fruits. Publicly available microarray data suggested that a subset of *VvWRKY*s was activated in response to diverse stresses. Quantitative real-time PCR (qRT-PCR) results demonstrated that the expression levels of 36 *VvWRKY*s are changed following cold exposure. Comparative analysis was performed on data from publicly available microarray experiments, previous global transcriptome analysis studies, and qRT-PCR. We identified 15 *VvWRKYs* in at least two of these databases which may relate to cold stress. Among them, the transcription of three genes can be induced by exogenous ABA application, suggesting that they can be involved in an ABA-dependent signaling pathway in response to cold stress.

**Conclusions:**

We identified 59 *VvWRKY*s from the *V. vinifera* genome and 15 of them showed cold stress-induced expression patterns. These genes represented candidate genes for future functional analysis of *VvWRKYs* involved in the low temperature-related signal pathways in grape.

## Background

Plants have a variety of defense mechanisms to protect themselves from adverse environmental effects. Families of transcription factors are involved in these processes by functioning to reorganize gene expression patterns. The WRKY family is among them and plays key roles in modulating genes expression during plant defense in response to pathogens [1,2]. The WRKY transcription factors were first identified in sweet potato (SPF1) as DNA binding proteins [3]. Two similar genes (*ABF1* and *ABF2*) were found in wheat during germination [4]. Subsequently, Rushton et al. [5] reported the identification and characterization of *WRKY1, WRKY2* and *WRKY3* from parsley (*Petroselinum crispum*) and proposed these genes belong to a gene family. This gene family was named WRKY due to a conserved region (WRKYGQK) that was identified in the N-terminal amino acid sequence of all the members [4,5]. Further studies showed that the conserved WRKY domain had other forms such as WRKYGKK and WRKYGEK [6], or the WRKY domain could be replaced by WKKY, WKRY, WSKY, WIKY, WRIC, WRMC, WRRY or WVKY [7,8].

According to variation in WRKY domain and a zinc finger motif in the C-terminus, WRKY proteins were divided into four groups [9,10]. WRKY proteins with two WRKY domains composed group I. Groups II and III were characterized by a single WRKY domain. Group II WRKY proteins were further subdivided into five or more subgroups based on short conserved structural motifs while group III proteins contained a variant zinc-finger which ends with HXC. Finally, group IV WRKY proteins contained the WRKY domain, but lack a complete zinc-finger structure in the C-terminus. WRKY proteins usually functioned as transcriptional regulators via binding to W-boxes (TTGACC/T) in the promoter regions of down-stream genes and clusters of W-boxes had an amplified effect [3-5,11-15]. However, some other studies have found that some WRKY proteins bind to the PRE4 element (TGCGCTT), SURE element (TAAAGATTACTAATAGGAA) or SURE-like element and the WK box (TTTTCCAC) [2].

WRKY proteins have been found to play essential roles in pathogen defense in response to bacteria [16,17], fungi [18,19], and viruses [20,21]. Evidence also supported that WRKY transcription factors were involved in modulating gene expression in plants during abiotic stresses such as cold [22,23], salt [24,25] and drought [26-28]. Besides roles in response to biotic and abiotic stress, WRKY proteins were also implicated in processes that modulate plant developmental processes such as morphogenesis of trichomes and embryos, senescence, dormancy, and metabolic pathways [2].

Grape is one of the most important fruit crops worldwide. The productivity of grapevines is largely limited by disease pressure and stressful fluctuations in environmental conditions. Due to their essential role in the early response to pathogens and abiotic stresses, several *WRKY* genes were intensively studied in grape. *VvWRKY1* and *VvWRKY2*, isolated from grape (*V. vinifera* cv. Cabernet Sauvignon) berries, were found to potentially participate in defending against fungal pathogens [18,29]. *VvWRKY1* was found involving in enhanced protection against *Botrytis cinerea* by transactivating the *VvLTP1* promoter [30], and *VvWRKY2* may regulate lignification and response to biotic or abiotic stresses in grapevine [31]. *VpWRKY1* and *VpWRKY2*, isolated from Chinese wild *V. pseudoreticulata*, may contribute to resistance to powdery mildew (*Erysiphe necator*) and tolerance to salt and cold stresses in grape [32]. *VpWRKY3* was found to be involved in pathogen defense and also interact with the salicylic acid, ethylene, and abscisic acid signal pathways [33]. Transgenetic *Arabidopsis* plants expressing *VvWRKY11*, isolated from ‘Beifeng’, an interspecific cultivar of *V. thunbergii × V. vinifera*, showed increased dehydration tolerance [34]. Its homologous gene, *VpWRKY11*, was found to serve as a negative regulator of disease resistance [35]. Although several individual *WRKY* genes have been identified in grapevine, the *WRKY* gene family in grapevine remains wholly uncharacterized.

Based on our previous transriptome analysis, we found that some *WRKY* genes respond to cold stress in different patterns in *V. amurensis* (a cold hardy grapevine species) and *V. vinifera* cv. Muscat Hamburg [36]. *VvWRKY14* (GSVIVT01015952001) and *VvWRKY12* (GSVIVT01012682001) were found up-regulated over 30 fold in *V. amurensis* after being subjected to cold stress but up-regulated to a lesser extent in *V. vinifera*. In contrast, the expression of *VvWRKY43* (GSVIVT01030258001) was up-regulated in *V. vinifera* (26 fold) while expression remained low in *V. amurensis*. These different gene expression patterns in response to cold stress may be contribute to the distinctive cold hardiness between the two species. To further characterize how *WRKY* genes respond to freezing stress of grapevine, we initiated this study to identify the entire *WRKY* gene family in grapevine based on the published 12× *V. vinifera* cv. Pinot noir (PN40024) genome sequences [37]. A phylogenetic tree was constructed for identified WRKY proteins and the gene expression patterns in different tissues of *V. vinifera* were detected by RT-PCR. *WRKY* genes responding to biotic and abiotic stresses were cross-evaluated by using public gene-chip databases. Additionally, real time RT-PCR was used to detect the expression level of *VvWRKYs* under cold treatment and exogenous ABA. A comparative analysis was conducted to identify *VvWRKYs* that may participate in cold signal transduction pathways in *V. vinifera* using microarray data in public databases, our previously reported transcriptome data and qRT-PCR analysis conducted in this study.

## Results

### Identifying of *WRKY* transcription factors in *V. vinifera* genome

A total of 64 transcripts in the *V. vinifera* genome sequence were identified as possible members of the WRKY family. Five transcripts were excluded due to a lack of the conserved WRKY domain in the predicted amino acid sequences. The remaining 59 transcripts were named from *VvWRKY1* to *VvWRKY59* according to their order in the *V. vinifera* genomic sequence (Table 1). As for the previously published six WRKY proteins in grapes [18,29-35], each amino acid sequence was downloaded and BLASTp was used to find its corresponding *WRKY* loci in the *V. vinifera* genome.

**Table 1:** **Identified *WRKY *** **genes in 12× *V. vinifera *** **‘Pinot Noir’ genome**

**Gene ID**	**Gene symbol**	**Subgroup**	**Chromosome no.**	**Peptide length**	**Related publications**
GSVIVT01000752001	*VvWRKY01*	IId	chr7	285	
GSVIVT01001286001	*VvWRKY02*	IV	chr2	106	
GSVIVT01001332001	*VvWRKY03*	I	chr1_random	436	*VvWRKY2*[29,30]
GSVIVT01007006001	*VvWRKY04*	I	chrUn	551	
GSVIVT01008046001	*VvWRKY05*	IIb	chr17	606	
GSVIVT01008553001	*VvWRKY06*	IIc	chr17	152	*VvWRKY1*[18]
GSVIVT01009441001	*VvWRKY07*	IId	chr18	320	
GSVIVT01010525001	*VvWRKY08*	IIc	chr1	190	
GSVIVT01011356001	*VvWRKY09*	IIb	chr14	503	
GSVIVT01011472001	*VvWRKY10*	I	chr14	890	
GSVIVT01012196001	*VvWRKY11*	IIc	chr1	284	
GSVIVT01012682001	*VvWRKY12*	IIb	chr10	511	
GSVIVT01014854001	*VvWRKY13*	I	chr19	623	
GSVIVT01015952001	*VvWRKY14*	IIa	chr9	279	
GSVIVT01018300001	*VvWRKY15*	IIc	chr15	229	
GSVIVT01019109001	*VvWRKY16*	I	chr4	487	
GSVIVT01019419001	*VvWRKY17*	IIe	chr2	324	
GSVIVT01019511001	*VvWRKY18*	III	chr2	343	
GSVIVT01020060001	*VvWRKY19*	IIb	chr1	595	
GSVIVT01020864001	*VvWRKY20*	IIc	chr12	312	
GSVIVT01021252001	*VvWRKY21*	IIe	chr10	279	
GSVIVT01021397001	*VvWRKY22*	IIc	chr10	320	
GSVIVT01021765001	*VvWRKY23*	IIe	chr10	422	
GSVIVT01022067001	*VvWRKY24*	IId	chr7	281	
GSVIVT01022245001	*VvWRKY25*	IIc	chr7	194	
GSVIVT01022259001	*VvWRKY26*	IIc	chr7	227	
GSVIVT01023600001	*VvWRKY27*	I	chr11	500	*VpWRKY2*[30]
GSVIVT01024624001	*VvWRKY28*	I	chr6	571	
GSVIVT01025491001	*VvWRKY29*	IV	chr6	122	
GSVIVT01025562001	*VvWRKY30*	I	chr8	439	
GSVIVT01026965001	*VvWRKY31*	IIe	chr15	349	
GSVIVT01026969001	*VvWRKY32*	IIc	chr15	202	
GSVIVT01027069001	*VvWRKY33*	III	chr15	361	
GSVIVT01028129001	*VvWRKY34*	IIe	chr7	243	
GSVIVT01028147001	*VvWRKY35*	IIc	chr7	303	
GSVIVT01028244001	*VvWRKY36*	IIb	chr7	480	
GSVIVT01028718001	*VvWRKY37*	III	chr16	365	
GSVIVT01028823001	*VvWRKY38*	IIe	chr16	183	
GSVIVT01029265001	*VvWRKY39*	IId	chr11	280	
GSVIVT01029688001	*VvWRKY40*	IIb	chr12	491	
GSVIVT01030046001	*VvWRKY41*	I	chr12	365	
GSVIVT01030174001	*VvWRKY42*	III	chr8	332	*VpWRKY1*[30]
GSVIVT01030258001	*VvWRKY43*	I	chr8	514	
GSVIVT01030453001	*VvWRKY44*	IIb	chr12	499	
GSVIVT01032661001	*VvWRKY45*	III	chr13	289	
GSVIVT01032662001	*VvWRKY46*	III	chr13	309	
GSVIVT01033063001	*VvWRKY47*	IIc	chr14	183	
GSVIVT01033188001	*VvWRKY48*	IId	chr4	268	*WRKY11*[33,34]
GSVIVT01033194001	*VvWRKY49*	IIc	chr4	157	
GSVIVT01033195001	*VvWRKY50*	IIc	chr4	102	
GSVIVT01034148001	*VvWRKY51*	IIc	chr8	300	
GSVIVT01034968001	*VvWRKY52*	IIc	chr5	310	
GSVIVT01035426001	*VvWRKY53*	IIc	chr4	167	
GSVIVT01035884001	*VvWRKY54*	IIa	chr4	263	
GSVIVT01035885001	*VvWRKY55*	IIa	chr4	287	*VpWRKY3*[32]
GSVIVT01035965001	*VvWRKY56*	I	chr4	531	
GSVIVT01036223001	*VvWRKY57*	IId	chr14	305	
GSVIVT01037686001	*VvWRKY58*	IIb	chr19	497	
GSVIVT01037775001	*VvWRKY59*	I	chr19	553	

The putative genome location of each *VvWRKY* in the grape genome was shown in Additional file 1: Figure S1. Fifty-eight of the *VvWRKY*s could be mapped to 18 of the 19 grape chromosomes, with no *VvWRKYs* found on chromosome 3. *VvWRKY4* was putatively located on the ‘Chromosome Unknown’. *WRKY* transcription factors were not evenly distributed across the chromosomes of the grape genome. There were most abundant on Chromosome 4 (8 *VvWRKYs*) and chromosome 7 (7 *VvWRKYs*) and least abundance on Chromosome 5 and 18 (1 *VvWRKY*).

### Categorization of VvWRKYs basis on conserved WRKY domains

**Figure 1:**
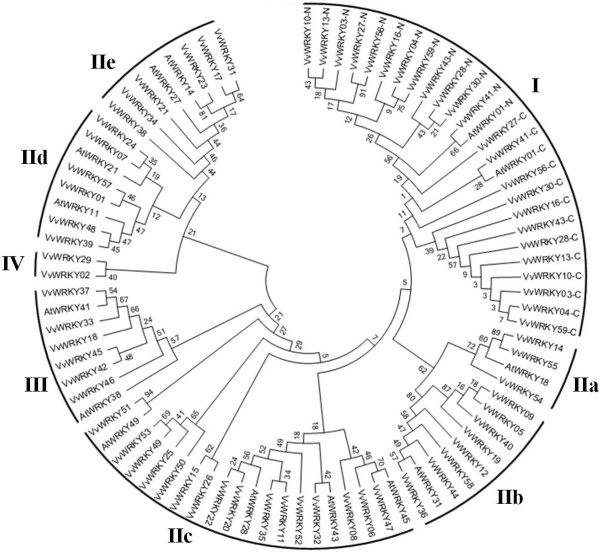
**Phylogenetic tree of *VvWRKY*s.** The unrooted phylogenetic tree of WRKY domains was constructed with MEGA5.1 program with the neighbor-joining method. The numbers beside the branches represent bootstrap values based on 1000 replications. The name of groups (I, II, III and IV) and subgroup (a–e) were shown at the outside of the circle. The WRKY named with suffix -N or -C indicated the N-terminal WRKY domain (NTWD) or the C-terminal WRKY domain (CTWD) in one VvWRKY with two WRKY domains. AtWRKY*s* were used as reference to categorize VvWRKYs.

The disposition of structural domains in amino acid sequences is an important clue to analyze the evolution and relationship between highly divergent sequences [38]. The relationships among the 59 WRKY proteins were investigated through constructing phylogenetic trees based on multiple alignments of the predicted amino acid sequences of the WRKY domains. As shown in Figure 1, we classified the 59 VvWRKY proteins into four large groups according to the results of the phylogenetic analyses. The models of conserved amino acid sequences of WRKY domain and zinc-finger structure in four groups were shown in Additional file 2: Figure S2. Twelve of the WRKY proteins contained two complete WRKY domains and a C_2_H_2_-type zinc finger motif. These proteins constituted group I. The N-terminal WRKY domain (NTWD) and C-terminal WRKY domain (CTWD) of VvWRKY27, VvWRKY41 and VvWRKY56 were clustered into a same clade in group I. According to Eulgem et al. [9] and by using WRKY proteins in *Arabidopsis* as references, 39 VvWRKY in group II were categorized into five subgroups. Three members were found in subgroup IIa, 8 in IIb, 16 in IIc, 6 in IId and 6 in IIe. Group II was divided into two parts. Subgroup IIa, IIb and IIc showed a close relationship with Group III WRKY proteins. And subgroups IId and IIe belonged to a separate clade which was closely related to group IV. Subgroup IIc showed higher divergence than the other subgroups. There were also 6 WRKY proteins in group III, and 2 in group IV which lacked a complete zinc-finger structure.

### RT-PCR based transcription levels detection of *VvWRKYs* in different tissues

**Figure 2:**
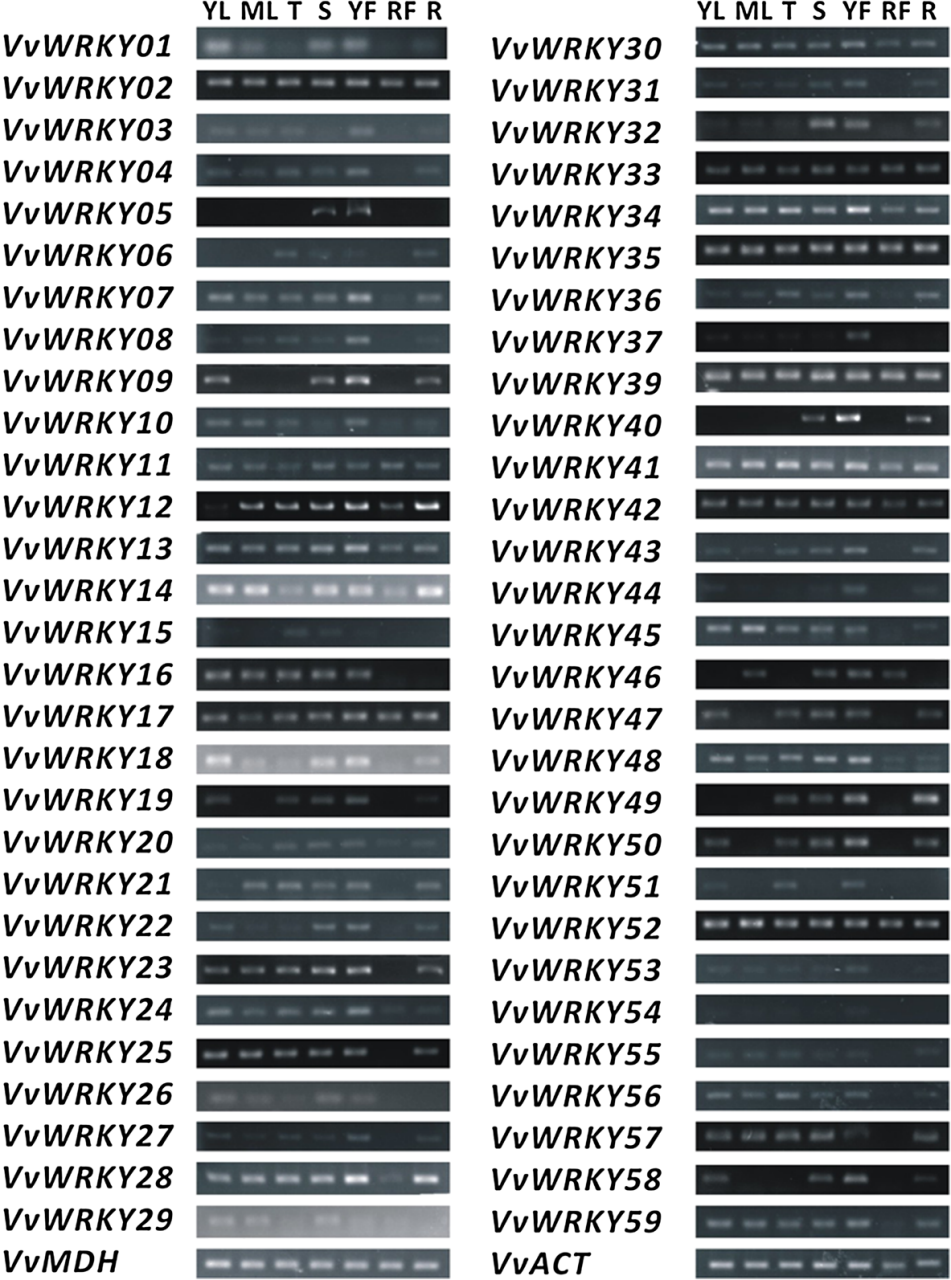
**RT-PCR analyses of presence of *VvWRKY *transcripts in seven grape tissues.** YL: young leaf; ML: mature leaf; T: tendril; S: stem apex; YF: young fruit; RF: ripe fruit; R: root. *VvMDH* and *VvACT* were used as control.

To investigate if the putative *VvWRKYs* were expressed and assess their transcription levels in grape, we examined the expression of these genes in different grape tissues. Among all *VvWRKYs*, we successfully designed and verified 58 primer pairs representing all candidate *VvWRKY*s except for *VvWRKY38* (Figure 2). All transcripts can be detected at least in one tissue. Nineteen *VvWRKY*s (including *VvWRKY02*, *11*, *12*, *13*, *14*, *17*, *20, 24, 28, 30, 33*, *34*, *35*, *36, 39*, *41*, *42*, *48* and *52*) were found expressed in all tissues used. Six *VvWRKYs* (*VvWRKY05*, *09*, *22*, *40, 44* and *58*) were found only expressed in young tissues. *VvWRKY05* was expressed in the stem apex and young fruit. *VvWRKY40* was found in stem apex, young fruit and root. *VvWRKY09*, *22*, *44* and *58* were detected in young leaf, stem apex, young fruit and root.

### Gene-chip based expression analysis of 26 *VvWRKYs* under various stresses

Although we identified *WRKY* transcription factors from the *V. vinifera* genome, functions for these genes in response to abiotic and biotic stress remain unknown. Using microarray results from publically available data, it was possible to find gene expression data from multiple experimental conditions for several of the grapevine *WRKY* genes. We carefully checked the genes on the ‘GeneChip *Vitis vinifera* (Grape) Genome Array’ (Affymetrix) and a total of 26 *VvWRKY*s were found on this chip. Microarray data related to salinity, water-deficit, PEG, cold, ABA and pathogen stresses were downloaded and their corresponding probes and the CV (coefficent of variation of the corresponding treatment means) of these genes in each of the microarray experiments were listed in Additional file 3: Table S1. If the expression of a probe set (gene) is affected by some of the treatments in an experiment, it shows a higher CV (more fluctuation); and vice versa. According to the data, the CV of 20 of the 26 *VvWRKYs* were over 5% in at least one experiment. The highest CV appeared in *VvWRKY57* (up to 36%) associated with compatible viral diseases in berry experiment in *V. vinifera* cv. Cabernet Sauvignon. *VvWRKY03*, *06*, *08*, *28*, and *55* responded to both abiotic and pathogens stresses while *VvWRKY21*, *39*, *48* seemed to respond primarily to pathogens stresses.

**Figure 3:**
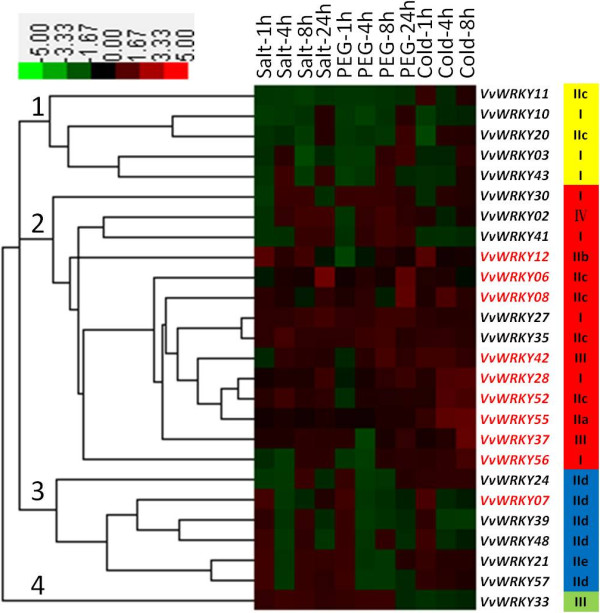
**Cluster analyses of *VvWRKY*s from 16 k Affymetrix *V. vinifera *gene-chip data in *PLEXdb *database.** The relative expression values of 26 *VvWRKYs* responding to different abiotic stresses (salinity, water deficit, cold) were used in analysis. Red, black and green elements in the matrix indicate up-regulated, no change and down-regulated WRKY genes, respectively. Those genes can be classified into four groups according to expression patterns, which were shown in different color with its group IDs that coincide with Figure 1. The red color was used to emphasize the *VvWRKYs* that changed in expression over 2 fold under cold treatment.

To test the correlation between the expression patterns of 26 *VvWRKY*s and their phylogenetic relationship, a hierarchical cluster analysis was performed using the 11 stress related experimental datasets (Figure 3). Red, black and green elements in the matrix indicate up-, no change- and down-regulated expression of *WRKY* transcription factors, respectively. From the heat map, twenty-six genes were clustered into four clades. Carefully analyzing the cluster of expression data in response to abiotic stresses experiments and comparing this with the VvWRKYs phylogenetic tree, we found that genes with close phylogenetic relationship were classified into the same clade during hierarchical cluster analysis. The most obvious evidence can be found in clades 3 with 5 WRKY subgroup IId genes (including *VvWRKY07*, *24*, *39*, *48* and *57*), which show similar expression patterns in response to salt, PEG and cold stresses. Clade 1 contained three WRKY group I genes and two group IIC genes. Clade 2 was mainly composed by WRKY group I and IIC and contains a majority of cold stress-related *VvWRKYs* (Also shown in Additional file 4: Table S2). Clade 4 only had one gene and that gene was from WRKY group III.

### Real-time RT-PCR based expression analysis of *VvWRKYs* under cold treatment in *V. vinifera*

**Figure 4:**
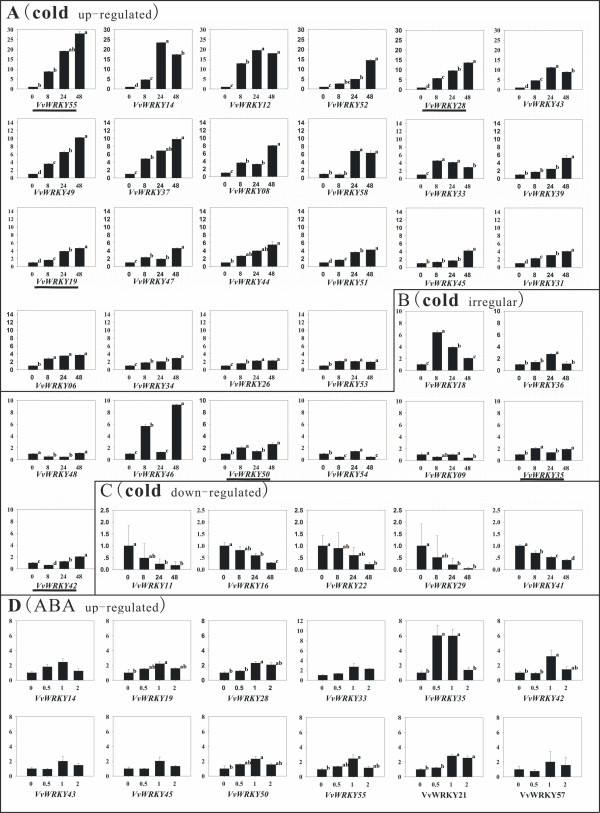
**qRT-PCR assays of the expression patterns of *VvWRKY*s under cold and exogenous ABA treatments.** The default expression value for each gene was 1 at 0 hours before treatment. A, B and C represent the subgroups with different expression patterns in cold treatment and D represents the genes that up-regulated over 2 fold in ABA treatment. **A**: sustained up-regulated genes in cold treatment; **B**: genes that changes over 2 fold but without significant tendency in cold treatment; **C**: sustained down-regulated genes in cold treatment; **D**: up-regulated genes in exogenous ABA treatment. *VvWRKYs* that accumulated in both cold and exogenous ABA treatments were underlined. One-Way ANOVA analysis was used to test the impact of timing of cold treatment. When the effects were significantly different, we examined the difference between treatments using post hoc multiple comparisons (LSD, *p* < 0.05). All data analyses were conducted using IBM SPSS Statistics 20, and the results were displayed through a, b, c and d.

To examine the response of *VvWRKY*s under cold stress in grape, we examined the transcription levels of *VvWRKY*s in shoot apices of ‘Muscat Hamburg’ under cold-treatment (4°C). *VvWRKY05*, *21*, *32* and *40* were excluded from cold-treated experiment since their Ct value of amplification curve were over 35 cycles in the templates of normal and cold-treated shoot apex. Detected *VvWRKYs* can be classified into four groups according to expression patterns as shown in Figure 4 and Additional file 5: Figure S3: A) sustained up-regulated during cold treatment (22 genes, Figure 4A), B) changed above 2 fold with irregular pattern (9 genes, Figure 4B), C) sustained down-regulated (5 genes, Figure 4C) and D) no significant difference (18 genes, as shown in Additional file 5: Figure S3). The relative expressions of 36 genes (Figure 4A, B and C) were significantly different as cold treatment. The greatest increase in expression (nearly 30 fold) was found in *VvWRKY55* at 48 h cold treatment. *VvWRKY18* and *VvWRKY46* had the largest up-regulation of greater than 6 fold at 8 hours after cold treatment. While *VvWRKY18* was degraded after 24 hours, the expression of *VvWRKY46* demonstrated both up and down regulated with a spike of expression at 48 hours after intensive degradation at 24 hours.

### Exogenous ABA induced accumulation of *VvWRKYs* in *V. Vinifera*

To illustrate how the *VvWRKYs* respond to ABA and whether the cold stress related *VvWRKYs* may participate in the ABA-dependent cold signal pathway, ABA treated grapevine apices were examined using qRT-PCR. *VvWRKY12*, *29*, and *46* were excluded from this experiment due to their higher Ct value (Figure 4D and Additional file 6: Figure S4). Among the 55 *VvWRKYs* we detected, twelve *VvWRKYs* were expressed over 2-fold greater within 2 h of exogenous ABA treatment (Figure 4D). After statistical analyses of qRT-PCR results, 7 of them were evaluated to significantly change during exogenous ABA treatments. Transcripts of *VvWRKY35* showed the greatest increase in expression at 0.5 h after ABA treatments. Six other genes showed increases in expression 1 h after exogenous ABA treatment (Figure 4D).

When the data from the cold and ABA experiments were compared, 6 of 7 genes (*VvWRKY, 19, 28, 35, 42, 50 and 55*) that were up-regulated during exogenous ABA treatment were also up-regulated under cold treatment (Figure 4A and B, marked by underline). Two genes (*VvWRKY55*, *28*) were greatly up-regulated, over 10 fold. The expression levels of the rest of the 44 *VvWRKYs* were lower than 2-fold and not significantly changed during exogenous ABA treatments (Additional file 6: Figure S4).

### Identification of candidate cold-stress related *VvWRKYs*

**Figure 5:**
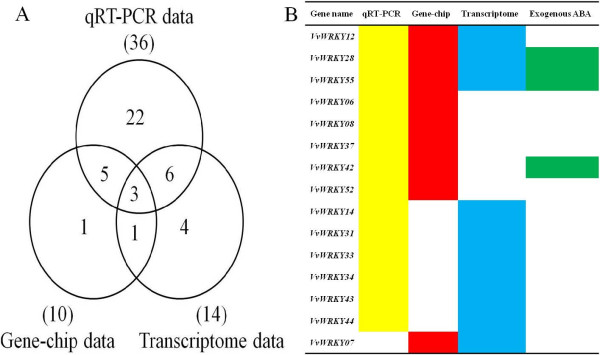
**An overview of cold stress-related *VvWRKYs *in three sets of data. A**: The Venn diagram of the cold stress-related *VvWRKYs* obtained from qRT-PCR, transcriptome and gene-chip data. **B**: The *VvWRKYs* that were found in more than one type of experimental data. Green color in forms indicated *VvWRKYs* induced by exogenous ABA.

Previously we reported the changes of the transcriptome during cold-treatments in ‘Muscat Hamburg’ and identified 14 cold-stress related *VvWRKYs* (we reported 16 *VvWRKYs* but subsequent annotation of these genes allowed us to exclude two genes that do not belong to the WRKY gene family)[36]. Gene-chip based methods also allowed to identify 10 cold-stress related *VvWRKYs*[39]. In order to overcome the deficiencies of determining gene expression from a single technological approach and obtain more reliable results, we compared the data from three different methods. Fourteen *VvWRKYs* from our previous transcriptome analysis, ten from publically available gene-chip based data and 36 genes from qRT-PCR results (this study) were used. The results were summarized in Figure 5 and Additional file 4: Table S2. Three *VvWRKYs* (*VvWRKY12, 28, 55*) showed identical expression patterns and were found up-regulated over 10 fold in at least one time-point under cold-treatment by qRT-PCR (Figure 4A). A total of 12 *VvWRKYs* were confirmed by two experimental methods (Figure 5A and B). *VvWRKY56* was identified as up-regulated gene under cold treatment only in the gene-chip studies. Twenty-two genes that were characterized by qRT-PCR were not supported by the other studies. It is worth mentioning that down-regulated *VvWRKYs* under cold-treatment were only identified by qRT-PCR based method.

## Discussion

### WRKY family in grape

Considering the important roles that *WRKY* transcription factors play during plant development and in response to various stresses, it is not surprising that we identified so many family members in grapevine. Previously, 74 *WRKY* genes were found in *Arabidopsis*[2], 55 in cucumber [40], 102 in rice [2], 47 in castor bean [41], 86 in *Brachypodium distachyon*[42] and 136 in maize [43]. Here we identified 59 candidate WRKY proteins in *V. vinifera* and categorized them into four groups.

### Group I WRKY proteins

When compared with WRKY family groups, WRKYs in primitive plant ancestors *Giatdia lamblia*, *Dictyostelium discoideum* and *Chlamydomonas reinhardtii* closely resembled *Vitis* group I [7,38]. In our study, two domains of VvWRKYs in group 1 were closely related. A BLASTp search of EuGene.1100010359 from an ancient alga species (*Ostreococcus sp. RCC809*) which has a single WRKY domain allowed us to identify 9 corresponding WRKY homologs in grape and 8 of these belonged to group I by MAP VIEW (Plant Genome Duplication Database) [44]. These data support the hypothesis that the dual WRKY domains present in members of group I may be derived from a single WRKY domain duplication [6,7].

### Group II WRKY proteins

Group II was divided into three parts: subgroup IIa + IIb, subgroup IIc and subgroup IId + IIe (Figure 1). Subgroup IIa + IIb belong to the same clade and is sister to the WRKYs in group I. Interestingly, the presumed function of CTWDs in group I for sequence-specific DNA binding [9] were more similar to the single WRKY domain members in group II and III than to the NTWDs of group I, This result may indicate that subgroup IIa + IIb evolved from group I by domain structure loss of the group I NTWD.

### Group III WRKY proteins

Group III in the phylogenetic tree was most closely related to the very large subgroup IIc, which was separately into four clades and seemed to indicate an expansion of the gene family. A thorough search of the Plant Transcription Factor Database (http://planttfdb.cbi.pku.edu.cn) indicated that the earliest evolutionary occurrences of group III genes were those found in ferns (*Selaginella moellendorffii*). There was no evidence of any sequenced plant species that only contain members of group I and III but we found in some species with only members of group I and II, for example in mosses (*Physcomitrella patens*) [1], and some gymnosperms (*Pinus taeda)* (http://planttfdb.cbi.pku.edu.cn). We speculated that group III may have evolved from group II, particularly IIc. As group III WRKYs in *Arabidopsis* responded to diverse biotic stresses [45], group III members may indicate adaptation of early plants to the stressful conditons associated with the colonization of land and subsequent increase in biotic pathogen pressures.

### Group IV WRKY proteins

We found that group IV WRKY proteins, which were characterized by the loss of the zinc-finger domain, were in the same clade as subgroups IId + IIe. *VvWRKY02* and *VvWRKY57* were duplicated gene pairs according to a whole genome analysis of grapevine gene duplications [46]. This might suggest an origin of group IV from subgroups IId + IIe. Group IV proteins were considered non-functional due to the loss of the zinc-finger domain [10]. However, these genes of group IV can be found in all higher plant species as well as in algaes (*Bathycoccus prasinos*: Bathy17g02050). Furthermore, some genes were expressed in rice (*OsWRKY56*) [10] as were two genes identified in this study (*VvWRKY02* and *VvWRKY29*). Therefore, it remains questionable whether group IV WRKYs have biological function in plants.

### *VvWRKYs* participate in development and stress-related signal pathways

*WRKY* genes were found to be expressed in many tissues and seem to be involved in regulating plant developmental and physiological processes. Transcriptomic analysis of senescence in the flag leaf of wheat demonstrated that *WRKY* transcription factors are greatly up-regulated during the senescence process [47]. *OsWRKY78* was found to be up-regulated in elongating stems and knockdown mutations in this gene cause plants to produce a semi-dwarf and small seed phenotype caused by reducing cell length [48]. Moreover, the transcription of *GhWRKY15* was observed abundant in the roots and stems of tobacco and transgenic overexpression lines of these plants displayed faster elongation at the earlier shooting stages [49]. Here the expression of 15 *VvWRKYs* (Figure 2) can be detected in all grape tissues we used, which may indicate its fundamental roles in different cell-types in grape. Similar to expression patterns observed in other plant species, *VvWRKYs* were found to be expressed in young tissues such as young leaf, shoot apex, tendril and young fruit.

Several numbers *of VvWRKYs* were found activated in more than one type of stress condition (Figure 3 and also Additional file 3: Table S1). *VpWRKY3*, homologous to *VvWRKY55* was observed to be up-regulated in response to many different sources of stress, including pathogen exposure, salicylic acid, ethylene, cold and drought stress [32]. *VvWRKYs* that were up-regulated in response to more than two types of stresses (e.g. pathogen and drought) supported the occurrence of cross-talk between signal transduction pathways in response to different stress conditions in plants [50].

Phylogenetic relationships between *VvWRKY* genes suggested that there may be conserved responses of these genes to salt exposure, PEG and cold-stress (Figure 3). All members of group IId clustered into one clade with similar expression pattern during these three stress conditions, suggesting the function of these VvWRKY proteins may relate to the structures of WRKY domains. Subgroup IId was identified as a novel CaM-binding transcription factor family in plants and their conserved structural motif was a Ca^2+^-dependent CaM-binding domain [51]. Thus the placement of the WRKYs in the phylogenetic tree may also help to predict function of new members that belong to certain gene family.

### *VvWRKYs* that participate in the cold related signal transduction in grape

Three different experimental methods were combined to robustly analyze the response of *VvWRKY* genes to cold stress (Figure 5 and Additional file 4: Table S2). Results from qRT-PCR demonstrated the greatest number of cold stress-related *VvWRKYs* (36) while gene-chip based methods identified the least, 10 *VvWRKYs*. This difference may be attributed to the method used but is also likely due to differences in the treatment conditions between experiments. During Digital Gene Expression profile (DGE) analysis [36], plant material was obtained from 4 h cold treatment at 4°C, whereas in our pRT-PCR experiment, we used samples collected at several different time periods (at 8 h, 24 h and 48 h after cold treatment at 4°C). Additionally, multiple matched tags were excluded from the final analysis performed by Xin et al. [36], which may have reduced the number of identified cold related *VvWRKYs.* Finally, gene-chip based methods may bias results due to a lower number of genes with corresponding probes related to the WRKY proteins (only 26 WRKY). By integrating the data from different methods, we obtained more reliable results and a total of 15 candidate cold tolerance *VvWRKYs* (Figure 5) were identified during our investigation.

According to previous studies, the transcriptional control of plant responses to cold stress can be divided into ABA-dependent and ABA-independent signal pathways [52]. The results of our study also indicated that 15 putative cold stress-related *VvWRKYs* can be divided into two groups according to their responses to exogenous ABA. Three *VvWRKYs* (*VvWRKY28*, *42* and *55*) may participate in an ABA-dependent signal pathway and other 12 in ABA-independent pathway. WRKY transcription factors have been identified as key components in the ABA signaling pathways [8,53]. In rice, *OsWRKY24*, *51*, *71* and *72* are induced by (ABA) in aleurone cells. *OsWRKY24* and *45* were functional as negative regulators in ABA induction of the *HVA22* promoter-beta-glucuronidase construct, while *OsWRKY72* and *77* synergistically interacted with ABA to activate this reporter construct [10]. It is still unknown how *WRKY*s participate in the cold stress-related signal pathway and what relationship these genes have with C-repeat Binding Factor genes (CBFs), which are critical transcription factors responsible for cold tolerance in plant [54].

The reliability of the identified 15 cold–related *VvWRKYs* was also supported by homologous genes in other species. *STHP-64*, which showed high similarity with *VvWRKY43*, was not present in leaves until November and December in *Solanum dulcamara*[55]. *WRKY38*, a homolog gene of *VvWRKY14*, was transiently accumulated when leaves and roots were exposure to low temperature in barley [56]. *BcWRKY46* showed higher similarity with *VvWRKY33* and responded to low temperatures in Pak-choi. Constitutive expression of *BcWRKY46* reduced the freezing susceptibility in transgenic tobacco [57]. The transcription level of *VvWRKY55* was up-regulated robust under cold treatment. Its homolog gene, *WRKY71* was found in banana with a similar expression pattern [58]. All these *VvWRKYs* mentioned above were confirmed by at least two set of experiment methods, which provided appropriate candidates to illustrate the roles of WRKY protein under low temperature-related signal pathways in grape.

Although low-temperature related *WRKY*s were isolated in several species, the mechanism of how *WRKY*s respond to cold signals and regulate the expression of downstream genes is still largely unknown. Further work is needed to elucidate the function of these important genes in low-temperature related signal pathways. Previously we reported the different expression patterns of *WRKY*s in *V. amurensis*, a cold-hardness species. The *WRKY* genes identified here from *V. vinifera* may accelerate the functional analysis of this gene family in *V. amurensis*. The comprehensive analysis of cold stress-related *WRKY*s in two different *Vitis* species with contrasting cold hardiness phenotypes would certainly help to illustrate the function of *WRKY* genes in conveying cold hardiness in grapevine.

## Conclusions

In summary, a total of 59 *VvWRKYs* in the *V. vinifera* genome were identified. The *VvWRKYs* were unevenly distributed in 18 of the 19 chromosomes. WRKY domain based phylogenetic analysis allowed categorizing 59 VvWRKYs into four large groups. A majority of *VvWRKYs* were found expressed in more than one tissue in *V. vinifera*. Gene-chip based data analysis suggested that a subset of *VvWRKYs* was activated in respond to diverse biotic and abiotic stresses. The transcription level of 36 *VvWRKY* genes changed over 2 fold after cold induction. A comparative analysis of qRT-PCR results, gene-chip based data and transcriptome analysis allowed us to identify 15 *VvWRKYs* that show identical expression patterns during cold treatment at least in two kinds of analyses. These studies not only increase our knowledge of WRKY family, but also provide candidate genes for future functional analysis of *VvWRKYs* involved in the low temperature-related signal pathways in grape.

## Methods

### Identification of *WRKY* genes in the grape genome

Candidate WRKY proteins were identified from the 12X *V. vinifera* cv. Pinot noir genome (quasi-homozygous line PN40024, http://www.phytozome.net). Full-length amino acids sequences of all WRKY proteins in *Arabidopsis thaliana* (http://www.arabidopsis.org/) were used as query sequences. A BLASTp search was performed and *E*-value of e^−6^ was used as the threshold [59]. Candidate WRKY proteins were manually confirmed [60] by searching for WRKY domains in the candidate amino acids sequences using SWISS-MODEL (http://swissmodel.expasy.org/) and the results were shown in Table 1.

### Phylogenetic analysis of WRKY family

Multiple alignments of the amino acid sequences of 73 WRKY domains from *V. vinifera* were performed using CLUSTALW by MEGA5.1 [61]. Twelve *Arabidopsis* WRKY domains from different WRKY groups were used as references to categorize the WRKY proteins from grape. The GenBank accession numbers of those AtWRKYs are AtWRKY01: ABJ17102, AtWRKY11: AEE85928.1, AtWRKY14: AAP21276.1, AtWRKY18: AAM78067, AtWRKY21: AAB63078.1, AtWRKY27: ABH04558, AtWRKY28: AEE84006, AtWRKY31: AEE84546.1, AtWRKY38: AED93044.1, AtWRKY41: AEE82969, AtWRKY43: AEC10646.1, AtWRKY45: ABD57509.1, AtWRKY49: AAQ62425.1. The parameters used during alignment were: protein weight matrix: Gonnet series; negative matrix: on; gap open penalty: 10; gap extension penalty: 0.20; delay divergent sequences: 30; residue-specific gap penalties: on; hydrophilic penalties: on; gap separation distance: 0; end gap separation penalty: on. An unrooted phylogenetic tree was constructed using Neighbor-Joining (NJ) methods and bootstrapped with 1,000 iterations to help identify WRKY protein groups.

### Plant materials

‘Muscat Hamburg’ (*V. vinifera*) was obtained from the Institute of Botany, the Chinese Academy of Sciences. Tissues of young leaves, mature leaves, tendril, stem apex, root, young fruits and ripe fruits were collected from the vineyard in July, 2012. Cold and exogenous ABA treatment experiments were performed on tissue culture seedlings of ‘Muscat Hamburg’ according to Li et al. [62]. Briefly, seedlings were cultured on 1/2 B5 medium with 30 g/L of sucrose in a growth chamber under 16-h light/8-h dark photoperiod at 26°C. Cold treatments were performed in another growth chamber with the same parameters except for temperature (4°C). Seedlings with five well developed leaves were used and the shoot apex with one well developed leaf was collected at 0 hour (h, used as control), 8 h, 24 h and 48 h. Seedlings with five well developed leaves were transplanted in 1/2 B5 nutrient solution. Exogenous ABA treatments were performed after one week under normal culture conditions with 100 μM ABA and the shoot apex with one well developed leaves were collected at 0 h (used as control), 0.5 h, 1 h and 2 h after treatments. Three independent replicates were collected for each time point and frozen in liquid nitrogen. Samples were then stored at - 80°C for the following RNA isolation.

### Expression patterns analysis of *VvWRKYs* by RT-PCR

Total RNA was isolated from collected samples using Plant Total RNA Isolation kit (Tiandz Inc; Beijing, China). RNase-free DNase (RQ1, Promega) was used to degrade DNA from total RNA. cDNA was synthesized by the SuperScript III Reverse Transcriptase (Invitrogen) with Oligo(dT)_18_ (Promega) according to the manufacturer’s instructions. Primer pairs (Additional file 7: Table S3) for *VvWRKY*s were designed by Primer 3 (http://bioinfo.ut.ee/primer3-0.4.0/) and tested by NCBI Primer BLAST. Two genes, β-actin (GenBank accession: EC969944; sense primer: 5′-CTTGCATCCCTCAGCACCTT-3′; antisense primer: 5′-TCCTGTGGACAATGGATGGA-3′) and malate dehydrogenase gene (MDH; GenBank accession: EC921711; sense primer: 5′-CCATGCATCACCCACAA-3′; antisense primer: 5′-GTCAACCATGCTACTGTCAAAACC-3′) were used as positive control for RT-CR [63]. Three biological replicate and 35 cycles for each reaction were performed. PCR products were detected by agarose gel electrophoresis with 2.5% gel concentration.

### Gene-chip based expression pattern analysis of *VvWRKYs*

We explored the expression profiles of *VvWRKY*s using publically available data from the 16 k Affymetrix *V. vinifera* gene-chip stored at *PLEXdb* (Plant Expression Database) [64] to explore the response of *VvWRKY*s during biotic and abiotic stresses in grape. The different studies and datasets that were included in these analyses were: A) a short term abiotic stress experiment in ‘Cabernet Sauvignon’ [39], B) a long-term salt and water stress study [65]; C) a study examining gene expression associated with compatible viral diseases in grapevine cultivars [66]; D) an experiment designed to examine the powdery mildew-induced transcriptome in a susceptible grapevine ‘Cabernet Sauvignon’ [67]; E) the complimentary dataset of the powdery mildew-induced transcriptome of a resistant grapevine ‘Norton’ [67]; F) a study of gene expression in grapevine in response to *Bois noir* infection [68]; G) a study of the grape skin transcriptome of berries grown on an exogenous abscisic acid treated vine [69]; H) the complimentary dataset of the grape skin transcriptome in the berries cultured in vitro and treated with exogenous ABA [69]; and lastly, I) a gene expression study associated with compatible viral diseases in the berry [70]. In our comparative analysis, we divided these experiments into either abiotic or biotic stresses related datasets. For each microarray experiment, the Affymetrix MAS5.0 normalized data were used for calculations of the fold change of differentially expressed genes. Probe sets corresponding to the putative *VvWRKY*s were identified at PLANEX (http://planex.plantbioinformatics.org) and completed via PLEXdb blast tool. Comparisons of *WRKY* expression level from gene-chip data for the short term abiotic stress treatment in ‘Cabernet Sauvignon’ was performed using Cluster 3.0 and JavaTreeview.

### Quantitative RT-PCR

Total RNA was isolated from cold and exogenous ABA treated shoot apices following the cDNA synthesis methods mentioned above. Synthesized cDNA was diluted 1:10 with ddH_2_O, and the quantitative RT-PCR reaction mixture contained 5 μl of 2 × SYBR Green I Master Mix (Roche, USA), 2.6 μL ddH_2_O, 0.2 μL of 10 μM solution of each primer and 2 μL diluted template cDNA. Reaction specificities for each primer pair was tested using qRT-PCR melting curve analysis. The experiment was carried out using a StepOnePlus real-time PCR Instrument (Applied Biosystems). Transcription levels of each *VvWRKY* was normalized against the average of β-actin, MDH (as mentioned above) and glyceraldehyde-3-phosphatedehydrogenase (GAPDH: CB973647; sense primer: 5′-TTCTCGTTGAGGGCTATTCCA-3′; antisense primer: 5′-CCACAGACTTCATCGGTGACA-3′) [63]. Each sample had three biological and two technical replicates to ensure the accuracy of results, and RNA samples with the same reverse-transcription (without Reverse Transcriptase) and dilution procedure were used as negative controls. The Ct values and the real-time PCR efficiencies were obtained using Lin-RegPCR [71] and the normalized relative quantities and standard errors for each sample were calculated by qbaseplus [72]. The relative expression level of each *VvWRKY* in different templates was calculated based on normalized relative quantities. We used One-Way ANOVA analysis to test the impact of timing of cold treatment. When the effects were significantly different, we examined the difference between treatments using post hoc multiple comparisons (LSD, *p* < 0.05). All data analyses were conducted using IBM SPSS Statistics 20.

## Abbreviations

ABA: Abscisic acid.

## Competing interest

The authors declare that they have no competing interests.

## Authors’ contributions

HPX, LNW, SHL and JPL designed and oversaw the research. LNW, LCF, XMS, LYS, ZCL and NW performed the research. LNW and WZ performed bioinformatics analysis, including gene identification and microarray data analysis. LNW, HPX, JPL and SHL wrote the article. All authors read and approved the final manuscript.

## Supplementary Material

Additional file 1: Figure S1 Chromosomal location of 57 *VvWRKY*s. *VvWRKY03* was located on ‘chromosome 1 random’ and *VvWRKY04* was located on ‘chromosome unknown’. Neither was shown here. Click here for file

Additional file 2: Figure S2 The models of conserved amino acid sequences of WRKY domain and zinc-finger structure in four groups. The numbers behind the charts indicated gene numbers in each group. Click here for file

Additional file 3: Table S1 The coefficient of variation of the corresponding treatment means (CV) and probe set IDs of *VvWRKY*s in 9 experiments. A higher CV means the expression of the probe set (gene) is affected by treatments in an experiment. Five *VvWRKYs* that didn’t show any changes in any treatments are marked by green color. Click here for file

Additional file 4: Table S2 Cold stress-related *VvWRKYs* obtained in one of three experimental methods. Yellow, red and blue forms represent genes obtained via qRT-PCR, gene-chip data and transcriptome data respectively. Exogenous ABA induced *VvWRKYs* were shown in green color in form. Click here for file

Additional file 5: Figure S3 Quantitative RT-PCR assays of the expression level of 18 *VvWRKY*s under cold treatment. The transcription level of these genes didn’t show significant changes during cold treatment in *V. vinifera.* Click here for file

Additional file 6: Figure S4 Quantitative RT-PCR assays of the expression patterns of 44 *VvWRKY*s under exogenous ABA treatment. The transcription level of these genes didn’t show significant changes during exogenous ABA treatment in *V. vinifera.* Click here for file

Additional file 7: Table S3 The primers used for expression pattern analysis for *VvWRKY*s. Click here for file
