# Supplementary figures and images for: Genome-wide identification of WRKY family genes and their response to cold stress in Vitis vinifera
Source: BMC Plant Biol. 2014 Apr 22;14:103. doi: 10.1186/1471-2229-14-103 (PMC4021059; doi:10.1186/1471-2229-14-103)

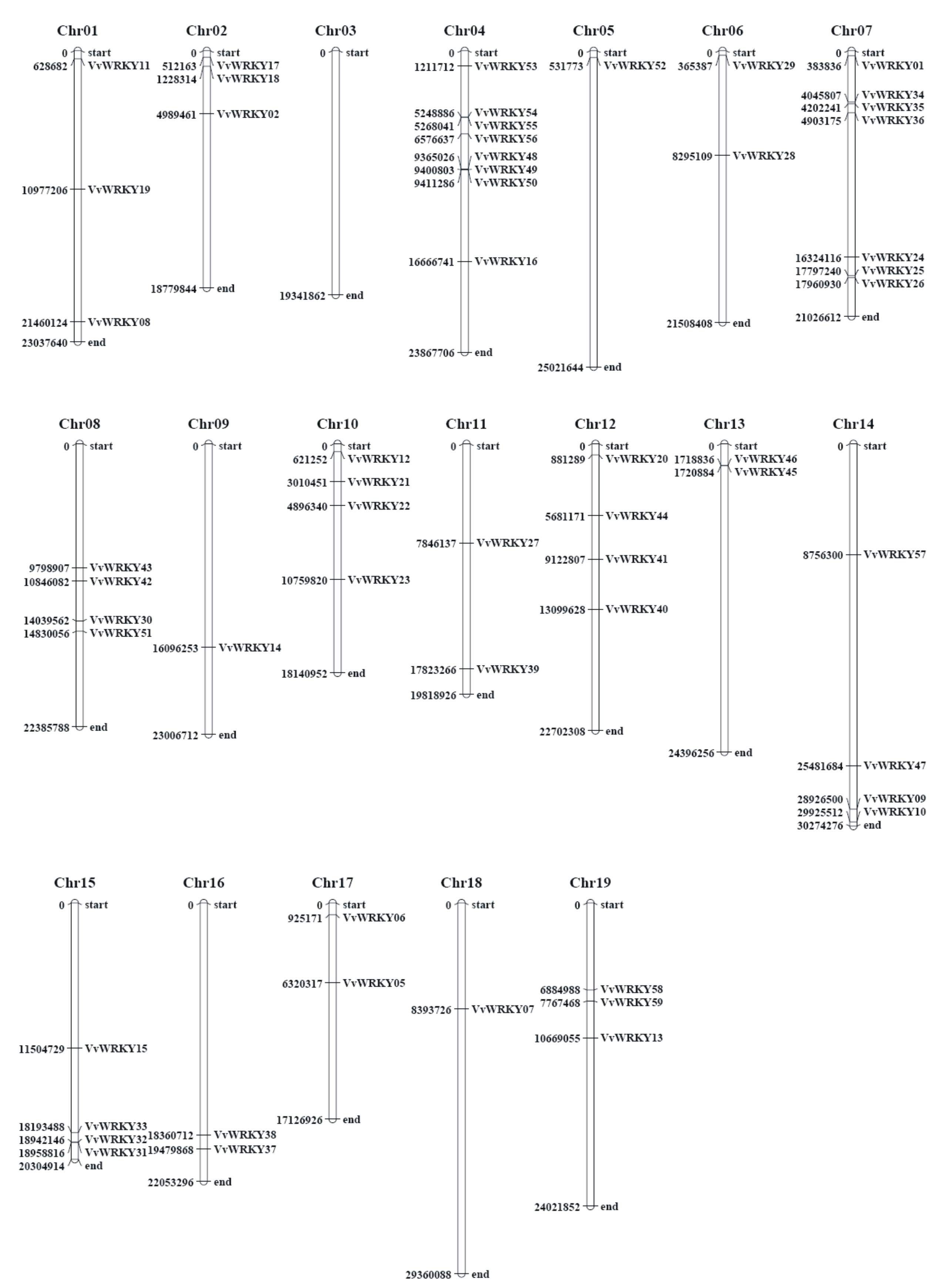

Supplement: Additional file 1: Figure S1 — Chromosomal location of 57 VvWRKYs. VvWRKY03 was located on ‘chromosome 1 random’ and VvWRKY04 was located on ‘chromosome unknown’. Neither was shown here. [file 1471-2229-14-103-S1.jpeg]

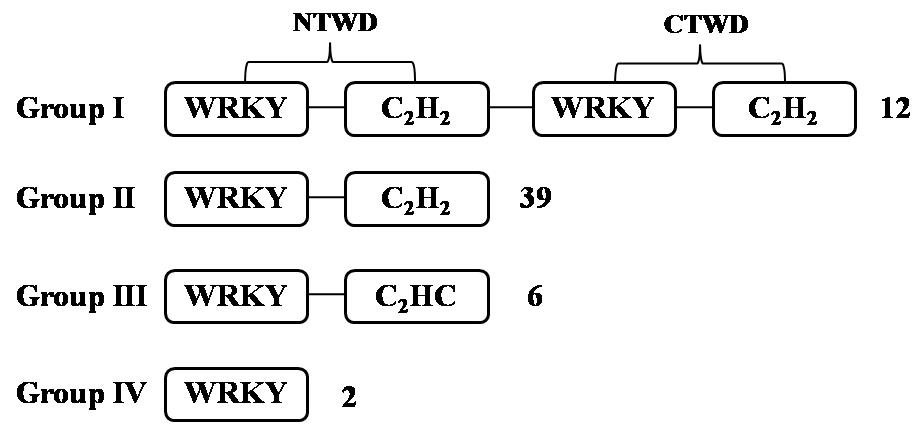

Supplement: Additional file 2: Figure S2 — The models of conserved amino acid sequences of WRKY domain and zinc-finger structure in four groups. The numbers behind the charts indicated gene numbers in each group. [file 1471-2229-14-103-S2.jpeg]

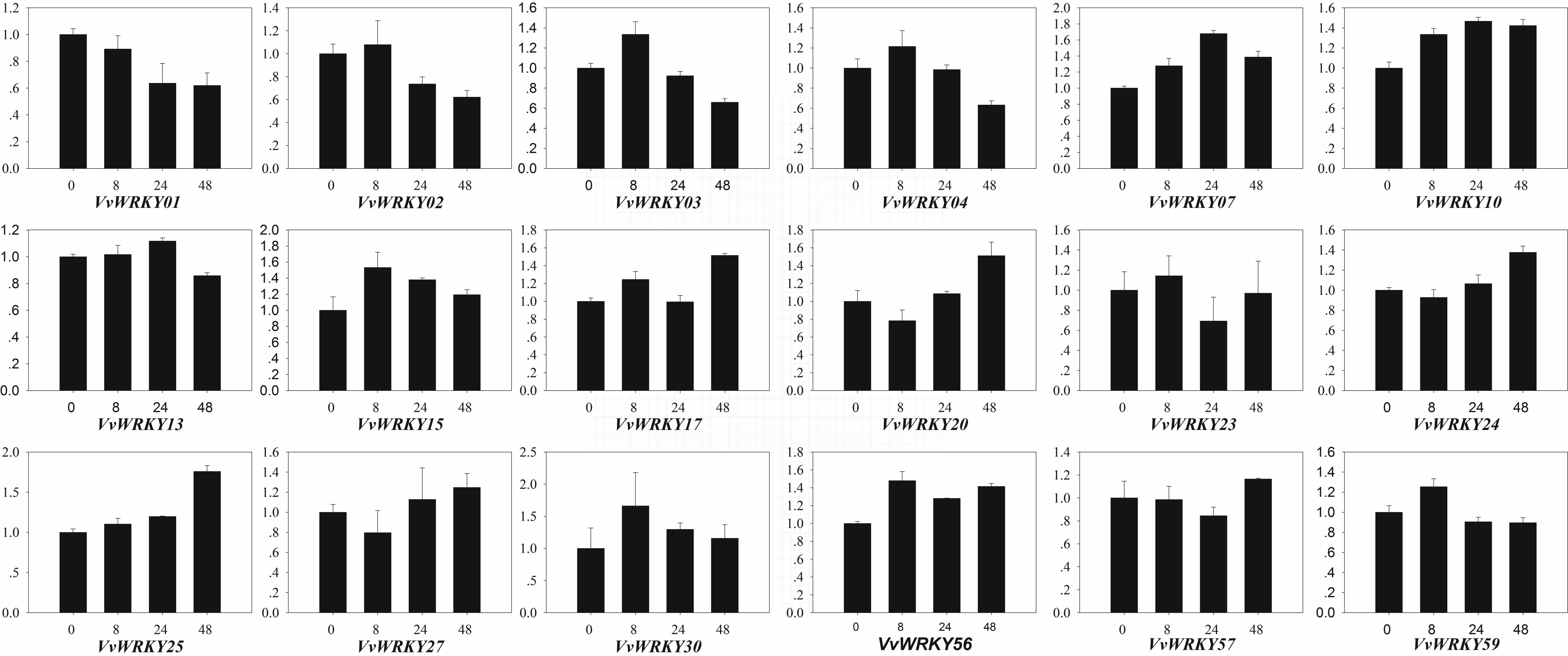

Supplement: Additional file 5: Figure S3 — Quantitative RT-PCR assays of the expression level of 18 VvWRKYs under cold treatment. The transcription level of these genes didn’t show significant changes during cold treatment in V. vinifera. [file 1471-2229-14-103-S5.jpeg]

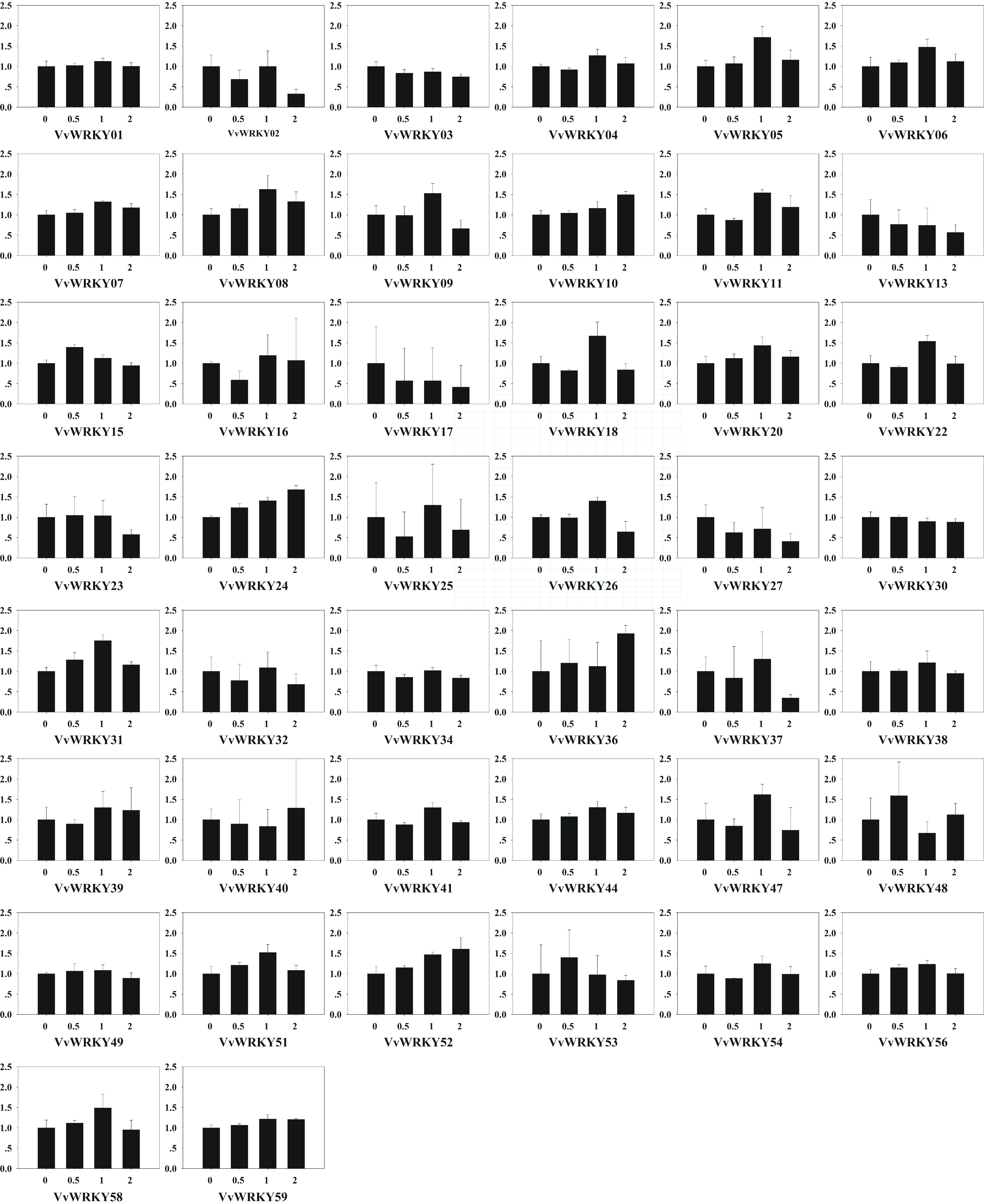

Supplement: Additional file 6: Figure S4 — Quantitative RT-PCR assays of the expression patterns of 44 VvWRKYs under exogenous ABA treatment. The transcription level of these genes didn’t show significant changes during exogenous ABA treatment in V. vinifera. [file 1471-2229-14-103-S6.jpeg]
