# Supplementary material for: Genome-wide identification of WRKY family genes and their response to cold stress in Vitis vinifera
Source: BMC Plant Biol. 2014 Apr 22;14:103. doi: 10.1186/1471-2229-14-103 (PMC4021059; doi:10.1186/1471-2229-14-103)
Supplement: Additional file 4: Table S2 — Cold stress-related VvWRKYs obtained in one of three experimental methods. Yellow, red and blue forms represent genes obtained via qRT-PCR, gene-chip data and transcriptome data respectively. Exogenous ABA induced VvWRKYs were shown in green color in form. [file 1471-2229-14-103-S4.docx]

Additional file 4 Table S2 Cold stress-related *VvWRKYs* obtained in one of three experiential methods. Yellow, red and blue forms represent genes obtained via qRT-PCR, gene-chip data and transcriptome data respectively. Exogenous ABA induced *VvWRKYs* were shown in green color in form.

| Gene name | qRT-PCR | Gene-chip data | Transcriptome data | Exogenous ABA |
| --- | --- | --- | --- | --- |
| *VvWRKY09* |  |  |  |  |
| *VvWRKY11* |  |  |  |  |
| *VvWRKY16* |  |  |  |  |
| *VvWRKY18* |  |  |  |  |
| *VvWRKY19* |  |  |  |  |
| *VvWRKY22* |  |  |  |  |
| *VvWRKY26* |  |  |  |  |
| *VvWRKY29* |  |  |  |  |
| *VvWRKY35* |  |  |  |  |
| *VvWRKY36* |  |  |  |  |
| *VvWRKY39* |  |  |  |  |
| *VvWRKY41* |  |  |  |  |
| *VvWRKY45* |  |  |  |  |
| *VvWRKY46* |  |  |  |  |
| *VvWRKY47* |  |  |  |  |
| *VvWRKY48* |  |  |  |  |
| *VvWRKY49* |  |  |  |  |
| *VvWRKY50* |  |  |  |  |
| *VvWRKY51* |  |  |  |  |
| *VvWRKY53* |  |  |  |  |
| *VvWRKY54* |  |  |  |  |
| *VvWRKY58* |  |  |  |  |
| *VvWRKY56* |  |  |  |  |
| *VvWRKY01* |  |  |  |  |
| *VvWRKY21* |  |  |  |  |
| *VvWRKY25* |  |  |  |  |
| *VvWRKY57* |  |  |  |  |
