# Supplementary material for: Genome-wide identification of WRKY family genes and their response to cold stress in Vitis vinifera
Source: BMC Plant Biol. 2014 Apr 22;14:103. doi: 10.1186/1471-2229-14-103 (PMC4021059; doi:10.1186/1471-2229-14-103)
Supplement: Additional file 7: Table S3 — The primers used for expression pattern analysis for VvWRKYs. [file 1471-2229-14-103-S7.docx]

Additional file 7 Table S3 The primers used for expression pattern analysis for *VvWRKY*s.

| Gene name | 3'primer | 5'primer | Product size |
| --- | --- | --- | --- |
| *VvWRKY01* | TTTGGAAGACCCTTCAATGC | TGGTTTGCAGATTGTGATGG | 81 |
| *VvWRKY02* | GGAGGAAATACGGGCAAAAG | ATGGATGGCCAGTCTGAAAG | 110 |
| *VvWRKY03* | GAAATGCCGCAAGTGAATCT | CTGGGGAACAAGCCTTCATA | 100 |
| *VvWRKY04* | ACAAACAATGGAGGCAGACC | ACGCTGATCAACGGAATCAT | 127 |
| *VvWRKY05* | TATGGCATACCATGCGAAGA | CCCATGAATATTTATCCACACG | 157 |
| *VvWRKY06* | TGTCCTTCATTTTGGAGGATCT | CGAACCCTTGACTCAAATGC | 142 |
| *VvWRKY07* | CACCCAATGGTGAATATCGTC | TGGCTAACATAGACACTCATCCTC | 108 |
| *VvWRKY08* | TCTTGAGCCAGATGCAAGTTT | GCCATTCCCCAATATCACAT | 140 |
| *VvWRKY09* | TCAGGCCATTTCTACCAACC | GGAGGAGGATGGAGTTTCCT | 110 |
| *VvWRKY10* | CCTTCTTGGCTGCTTGTTTC | AGAGACGATTCCAGGGTGCT | 118 |
| *VvWRKY11* | TGTCTACTTCTTCGGCAGTGA | CAGATTCCACCCAAACTCG | 61 |
| *VvWRKY12* | GTGGGAGGGTTAGCACACAG | GCATTGGACTTCGTTGATGA | 146 |
| *VvWRKY13* | GTTGCCAAAAGGAGAACCAA | AAGCGGTAGCCTGCTCATAA | 103 |
| *VvWRKY14* | AGAAAAAGGAAGGCAGAGAGC | AGTTCCCAACGACTCCATTGT | 61 |
| *VvWRKY15* | TGCTCGACGATGGTTACAAA | TCCACCCTCTTCTTCACTCG | 118 |
| *VvWRKY16* | GTCTGCCTGGCTTTGACTTC | TGCATCACCAATGTCATCCT | 140 |
| *VvWRKY17* | CCTGGAGGACATGGACTTTT | GTCGTGTGTGGTGGAATCAG | 101 |
| *VvWRKY18* | TGCACCTACAGAAACCTCCAC | CTTCATCCGAACGCTGAACT | 61 |
| *VvWRKY19* | CAAGGGAGAAAACCCTAGCC | GCAGAAGAGGAACGGTTCAA | 123 |
| *VvWRKY20* | AACATTACTCCCCACCACCA | TCGGTTCATCTACCATGCAA | 140 |
| *VvWRKY21* | CTTCTACGTCTTCCCCCAAAC | GGCACTGACACCACTCTCTTT | 61 |
| *VvWRKY22* | TGGCTTGTCACCTTCATCCT | GACTGGCTTATGATTGCCTTC | 61 |
| *VvWRKY23* | ATGGGACAGAAAGCTGAGGA | TGAGAGAAAGGGCGAGAGTC | 120 |
| *VvWRKY24* | CAACTCCATGCTCTCCCTGT | CCAACTCCACCATTTCCTTC | 113 |
| *VvWRKY25* | TGAGTTATGTGGGGATGGTG | CCAGCCCATGCGTACTTAG | 103 |
| *VvWRKY26* | GAAGAGTCGCAAGCTCCATC | CATAAGCTCGGGGGAATCTA | 122 |
| *VvWRKY27* | TCCGATGGTTGGTTCTGAAT | GTCTGTTTCCTCCGACTTGC | 141 |
| *VvWRKY28* | GAACCAAAGAAGAGCCCAGAG | GCAGAGCTTCAACAGAGCAGA | 61 |
| *VvWRKY29* | ATCAACAACCCCACTTCACAC | GGCTTCTCAGCAACAGAAAGA | 61 |
| *VvWRKY30* | TGATAGCGCTGTGAAAATCG | CCCAGGGAAAAAGATAGGATG | 137 |
| *VvWRKY31* | ATGGGTTTTGGAAGCATTTG | GGAAAAGCAATGGAAGTCTTTTT | 113 |
| *VvWRKY32* | GGCAGCATCAAGGATAGGAG | TTCCTCCAGCGATATCCATC | 119 |
| *VvWRKY33* | CCAACTATTTTCCGGTGTCG | GACTGAAGTTGGGGCTGAGA | 110 |
| *VvWRKY34* | GCCAGTGATGAAGATGCAAGT | CATGGCTGATTGTGGATTG | 61 |
| *VvWRKY35* | GCATGTAATTCGCTTGCTGA | TCCCTCCATTGGATCAAAAA | 134 |
| *VvWRKY36* | CATGCATGTGTTTTCAGGCTA | TCGAATGCTTGTGAGCAAAC | 112 |
| *VvWRKY37* | GCATATCCATATGTATGCAGCAG | ACCAAAATTGGCAGGTGAAG | 130 |
| *VvWRKY38* | ACCACACCATTGACTGCTCA | CGTCTTCAAACATTGGCTCA | 91 |
| *VvWRKY39* | TGCTCATCGTCACCTACGAG | TGTGACAACTTGCTCCCTGA | 138 |
| *VvWRKY40* | AATCCCGACTTTAGGGTTGC | AGAACCCAGTTGTTGCCACT | 149 |
| *VvWRKY41* | CATGCCCTCCATCATACCTG | CCCATACTTTCGCCAGTTGTA | 61 |
| *VvWRKY42* | ATGATCAGGAGCTCACCACA | TCATCCAAACTGTGAGTGCTG | 106 |
| *VvWRKY43* | AAAGCAAAGTGGTGGGTCTG | GGGCCTCAAAAACAAATTGA | 128 |
| *VvWRKY44* | CAATAACGCCAACACCACAG | CAGTTCCCTGGAAATTTTGAA | 114 |
| *VvWRKY45* | GTGCTTGGGTGTCTGGATTT | AACCTCTGGGGGTTTTCTACA | 118 |
| *VvWRKY46* | TGGGATCAAAGTACCCAAGG | TGTGGCAGGTGTGATCATCT | 141 |
| *VvWRKY47* | GATGTACCCATCAGGGATGC | GGCTTCTCGATTGGATGTGT | 112 |
| *VvWRKY48* | AGGAGTGGGCTTGGTGTTC | GGGACCACCTCTTCACTCAA | 147 |
| *VvWRKY49* | AGGACGAAATCAGAGCTGGA | TGGCAGTCTCCACTTGAACA | 122 |
| *VvWRKY50* | ATAGATGTTCAAGCAGAGGATGC | GAGTCATCCCTATCCCTTTCTACC | 61 |
| *VvWRKY51* | CACAAGGGTTGCTGGAAGAT | GCGAGCAAGAAGGAGAAGAA | 129 |
| *VvWRKY52* | CCACAGCCTCAGTATCAGCA | CATGGAGGAAAGTGGGGTTA | 109 |
| *VvWRKY53* | GGACTCACCCACAATTTGCT | GAATGAATTGGCTATCCTGCTC | 103 |
| *VvWRKY54* | AAGGAGCACACAACCATGAAC | GGTGAATATGAGGAACCACCTG | 61 |
| *VvWRKY55* | AGCCATTTCCGGAAGAATTT | ATCTGGGAATGAACCAAGCA | 110 |
| *VvWRKY56* | AGTCCACGAACTGCTGCTTT | CAGCTGACTGCTGGTTTGAA | 112 |
| *VvWRKY57* | CAACCAAAAGCTGGTTGATGT | TGATTGCATGTCTGAACCAAA | 144 |
| *VvWRKY58* | TCAATCCAGTGCTTCACAGC | AGGATGATGACGATGGCTTC | 111 |
| *VvWRKY59* | TGCAATTGCCTTCTGTGTCT | GAGACCTTCCATTTCCAGCA | 150 |
